# Effect of potent inhibitors of phenylalanine ammonia-lyase and PVP on in vitro morphogenesis of *Fagopyrum tataricum*

**DOI:** 10.1186/s12870-025-06440-x

**Published:** 2025-04-15

**Authors:** Reneé Pérez-Pérez, Artur Pinski, Magdalena Zaranek, Manfred Beckmann, Luis A. J. Mur, Katarzyna Nowak, Magdalena Rojek-Jelonek, Anna Kostecka-Gugała, Przemysław Petryszak, Ewa Grzebelus, Alexander Betekhtin

**Affiliations:** 1 Institute of Biology, Biotechnology and Environmental Protection, Faculty of Natural Sciences, University of Silesia in Katowice, Katowice, Poland; 2 Institute of Biological, Environmental and Rural Sciences, Aberystwyth University, Penglais Campus, Aberystwyth, Wales SY23 2DA UK; 3 Department of Plant Biology and Biotechnology, Faculty of Biotechnology and Horticulture, University of Agriculture in Krakow, Ave. Mickiewicza 21, Krakow, 31 - 120 Poland

**Keywords:** 2-aminoindan- 2-phosphonic acid (AIP), Fagopyrum, In vitro cultures, Polyvinylpyrrolidone, Phenylalanine ammonia-lyase, Lyase

## Abstract

**Background:**

*Fagopyrum tataricum* (Tartary buckwheat) is known for its high phenolic content, particularly rutin. High concentrations of these compounds secreted in the tissue culture medium can lead to its darkening and the eventual death of explants in in vitro cultures. This study aims to enhance the morphogenesis of *F. tataricum* callus cultures by utilising phenylalanine ammonia-lyase (PAL) inhibitors and polyvinylpyrrolidone (PVP) to mitigate oxidative browning and improve tissue viability.

**Results:**

We analysed the response of protoplasts isolated from morphogenic callus to media supplemented with varying concentrations of PAL inhibitors (AIP, AOPP, OBHA) and PVP. The flow cytometry results revealed that 10 µM AIP and 1% PVP yielded exclusively diploid plants, whereas higher concentrations (100 µM AIP and 3% PVP) failed to regenerate plants. Moreover, AOPP and OBHA addition resulted in the regeneration of tetraploid plants. Further analysis of proembryogenic cell complexes (PECCs) isolated from Tartary buckwheat morphogenic calli responses to AIP and PVP indicated that 100 µM AIP was most effective for plant regeneration. Metabolomic analysis showed that AIP treatments reduced phenolic compounds, notably rutin, and increased the GSH/GSSG ratio, indicating reduced oxidative stress. Gene expression analysis highlighted elevated expression of somatic embryogenesis-related genes (*LEC2*, *BBM*) and *WUSCHEL* in AIP-treated callus.

**Conclusions:**

This study demonstrates that AIP enhances the regeneration potential of *F. tataricum* callus cultures, offering valuable insights for optimising tissue culture techniques for industrial crops. Additionally, we have detailed the metabolomic changes in calli treated with PVP and AIP, highlighting their impact on metabolism.

**Graphical Abstract:**

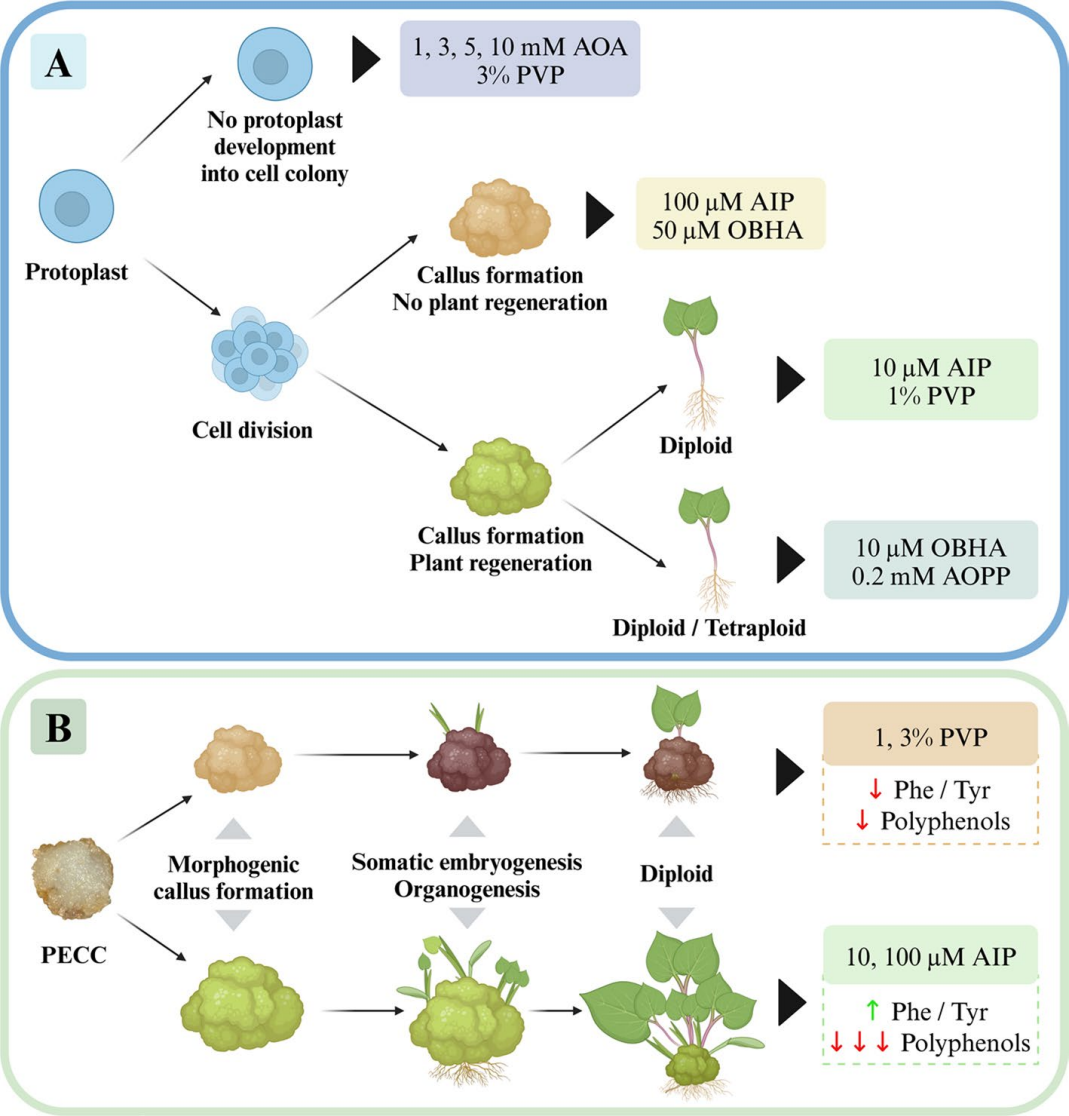

**Supplementary Information:**

The online version contains supplementary material available at 10.1186/s12870-025-06440-x.

## Introduction

The genus *Fagopyrum*, commonly known as buckwheat, comprises several species, among which *Fagopyrum esculentum* Moench. (common buckwheat) and *Fagopyrum tataricum* (Tartary buckwheat) are the most cultivated. Both species are known for their nutritional value, particularly high content of proteins, fibers, vitamins, and phenolic compounds such as flavonoids [32]. The major flavonoid present in buckwheat is rutin, with a concentration more than 100-fold higher in Tartary, compared to the common buckwheat [38]. Generally, the production of phenolic compounds in Tartary buckwheat is considerably higher [59] and their levels depend on the stage of development, or cultivar [28].

The improvement of Tartary buckwheat callus cultures is important in developing agronomically relevant traits, as they allow for efficient *Agrobacterium*-mediated transformation, protoplast isolation and subsequent plant regeneration [54, 65]. Calli induced from immature embryos of Tartary buckwheat can regenerate entire plants via morphogenesis (morphogenic callus, MC), which occurs either through somatic embryogenesis (formation of somatic embryos) or organogenesis (in the case of Tartary buckwheat only shoots). These calli exhibit genetic stability, crucial for effective propagation, and retain the morphogenic potential over prolonged periods of culture despite possible changes in their morphology [5, 64]. Tartary buckwheat MC consists of proembryogenic cell complexes (PECCs) and a ‘soft’ callus. According to histological studies, PECCs are embryos arrested at the preglobular stage by supplementing the culture medium with auxin. The PECCs contain phenolic-containing, meristematic and parenchymatous cells. During the cyclical callus development, the mature PECCs disintegrate, giving rise to young PECCs and ‘soft’ callus cells. Phenolic-containing cells separate from the PECCs and, as they become senescent, accumulate phenolics until the disruption of the tonoplast leads to their release in the cell and the culture medium. The ‘soft’ callus is formed during the loosening of the PECCs and consists of elongated cells that are highly vacuolated, metabolically active, but unable to divide; and whose function is to support the growth of the PECCs by providing sugars, proteins, and other conditioning factors. Due to their high phenolic content, Tartary buckwheat callus cultures are prone to browning. This phenomenon leads to medium darkening and can subsequently result in the death of the explants, thus limiting the success of in vitro plant genetic manipulation and multiplication [5].

The biosynthesis of phenolic compounds is controlled by a key enzyme, phenylalanine ammonia-lyase (PAL), that catalyses the deamination of phenylalanine to trans-cinnamic acid [23, 35]. Inhibitors of PAL like ɑ-aminooxyacetic acid (AOA), ɑ-aminooxy-β-phenylpropionic acid (AOPP), 2-aminoindan- 2-phosphonic acid (AIP), O-benzylhydroxylamine hydrochloride (OBHA) have been shown to reduce the production of phenylpropanoids thus limiting the cell wall lignification and increase the susceptibility of plants to pathogens [57]. Cinnamic acid and following *p*-coumaric acid are the precursors for various phenolics that are substrates for the polyphenol oxidase (PPO) and peroxidase (POD) [12]. These enzymes catalyse the oxidation of phenolics to quinones, which undergo non-enzymatic polymerisation with amino acids and proteins forming brown or black pigments known as melanins [34], resulting in oxidative browning [6, 52]. The melanins disrupt tissue metabolism, inhibit growth, and can lead to the death of the explants [24, 64]. The *p*-coumaric acid can also be produced by tyrosine ammonia-lyase (TAL) by deamination of tyrosine [63]. However, the activity of TAL is usually much lower in most plants compared to PAL [15]. Therefore, any disruption in PAL activity typically results in noticeable phenotypic changes, including decreased levels of phenolic compounds and associated changes in plant growth, development, and stress responses [8, 15].

The most common strategy to avoid tissue damage by phenolic accumulation is frequent transference of the explants to a fresh medium [40]. Other approaches include adjusting growth conditions, pre-treating explants, or supplementing the culture media with specific additives [31, 39, 46]. Most of those additives can be grouped into three categories: i) antioxidants, which is attributed to the capacity of the phenolics to inactivate free radicals, donating hydrogen atoms or electrons (i.e. ascorbic acid, citric acid, glutathione) [21, 56]; ii) adsorbents, that bind phenolics due to different kinds of interactions like hydrogen bonding and π-π interactions, as well as hydrophobic interactions (i.e. activated charcoal, PVP, PVPP) [33, 41], and iii) inhibitors, which work by targeting and neutralising the enzymes or intermediates involved in the browning process (i.e. tropolone and cysteine – PPO inhibitors, salicylhydroxamic acid – POD inhibitor, phosphonic analogues of phenylglycine – PAL inhibitors) [22, 48, 58].

Polyvinylpyrrolidone (PVP) was the first polymer used against oxidative browning and nowadays remains the first option in the tissue culture [9]. The repeating unit of PVP is a five-membered lactam ring (pyrrolidone) attached to a vinyl group. Phenolic compounds bind to PVP primarily through hydrogen bonding but can also occur by hydrophobic interactions [3]. Hydrogen bonding between the carbonyl group of the pyrrolidone ring and the multiple hydroxyl groups of phenolic compounds is particularly strong [40]. PVP can also interact with amino acids, proteins, and other secondary metabolites, but its affinity for those compounds is generally lower than phenolic compounds [51]. In in vitro cultures, PVP has been shown to reduce phenolic compounds and promote callus regeneration in tree peony. Transcription factors such as MYB, bHLH, and WRKY were shown to play a role in callus browning as indicated by transcriptome analysis [17]. Further analysis indicated that the combined use of activated carbon and PVP promotes rooting development by enhancing the expression of genes related to cytochrome P450 and phenylpropanoid biosynthesis [11]. However, PVP can also negatively impact the growth of tissue cultures, necessitating a more targeted approach, such as the use of inhibitors. AIP is a synthetic phosphonic analogue of phenylglycine acting as a PAL inhibitor [24, 27]. The indan ring in AIP resembles the aromatic ring of phenylalanine, fitting into the active site of PAL. Alternatively, the phosphonic acid group mimics the carboxyl group of the amino acid but is more electronegative and forms strong hydrogen bonds and ionic interactions with the active site residues, creating a more stable and less likely to dissociate bound complex than the natural substrate [58]. The AIP–PAL binding is often considered irreversible [4] and AIP significantly reduces browning and enhances the viability of callus cultures [23, 29, 37]. Besides the in vitro cultures, the application of AIP in rice plants reduced infection by the root-knot nematode *Meloidogyne graminicola*. Transcriptomic and metabolomic analyses indicated that AIP activates the phenylpropanoid pathway, thereby enhancing defence in rice through the activation of jasmonate-mediated defence mechanisms [36]. In *A. thaliana* plants, reactive oxygen species regulate fumonisin B1 (FB1)-induced cell death with AIP found to inhibit FB1-induced lesion formation and prevent the increase in salicylic acid levels [61].

This study aimed to use protoplast-derived cultures as a model system to evaluate the full range of PAL inhibitors and phenolic adsorbents by assessing their effects on plating efficiency (the number of cell colonies in one-week-old protoplast-derived cultures) and regenerative capabilities. We assessed the structural, genetic, and metabolic impacts of the AIP inhibitor and adsorbent PVP to establish the most valid approach to improved Tartary buckwheat callus culture.

## Materials and methods

### Plant material

The MC derived from an immature zygotic embryo of *F. tataricum* (L.) Gaertn was used to assess PAL inhibitors and phenolic adsorbent effects on protoplast-derived cultures’ plating efficiency and regenerative capabilities. The callus was maintained in the dark at 25 ± 1 °C on RX medium [5, 45] (Supplementary Table S1, sheet 1).

### Protoplast-derived cultures and plant regeneration

**Fig. 1:**
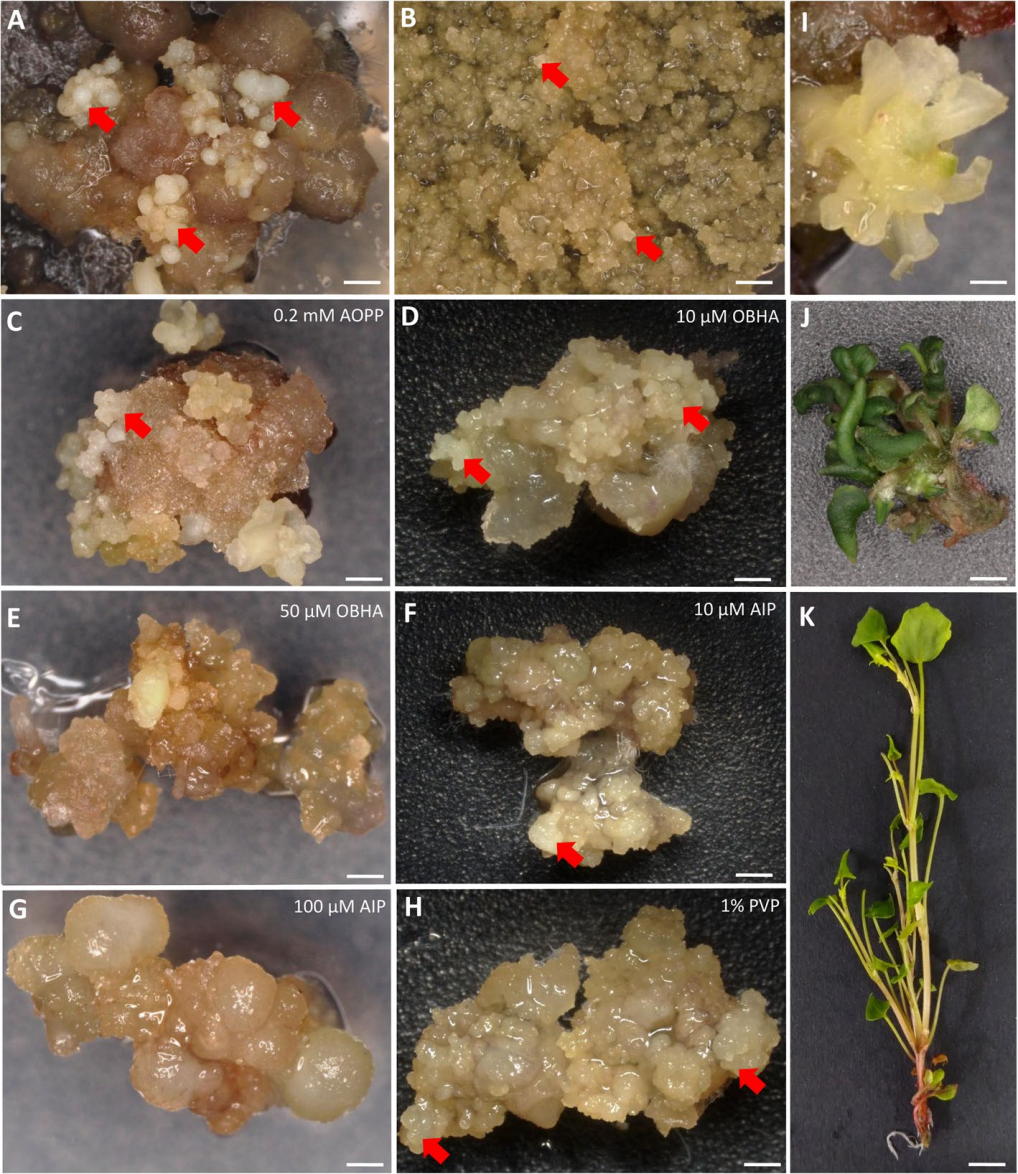
Lines of morphogenic callus (MC) of *F. tataricum* used for protoplast isolation: L1 (**A**), L2 (**B**). Callus obtained from MC-derived protoplast cultures, protoplast-derived cultures variant: 0.2 mM AOPP (**C**), 10 µM OBHA (**D**), 50 µM OBHA (**E**), 10 µM AIP (**F**), 100 µM AIP (**G**), 1% PVP (**H**). Somatic embryos (**I**), multiple shoots (**J**) and protoplast-derived plants (**K**). Red arrows indicate pro-embryogenic cell complexes. Scale bars: 1 mm (**A**–**H**), 5 mm (**I**–**K**). Abbreviations: AOPP – α -aminooxy-β-phenylpropionic acid; OBHA – O-benzylhydroxylamine hydrochloride; AIP – 2-aminoindan- 2-phosphonic acid; PVP – polyvinyl pyrrolidone

Protoplasts were isolated from two lines of *F. tataricum* MC, 12-day-old line L1 (Fig. 1A) and 7-day-old line L2 (Fig. 1B) according to the protocol established by Zaranek et al. [64]. Protoplasts were cultured on basal medium supplemented with 100 nM phytosulfokine-α (PeptaNova GmbH) and different concentrations of phenolics inhibitors: 1 mM, 3 mM, 5 mM, 10 mM of AOA; 0.2 mM of AOPP; 10 µM or 50 µM of OBHA; 10 µM or 100 µM of AIP; and 1% or 3% of the adsorbent PVP. The mechanism of action of AIP and PVP are shown in Supplementary Figures S1 and S2. After 10 days of culture, the medium, with additional supplements, was renewed. Agarose beads overgrown by microcalli were transferred to a callus multiplication medium (CM) [64] enriched with the same additives as in the protoplast-derived cultures. Each variant of the callus culture was maintained at 26 ± 1 °C in the dark and subcultured every three weeks. After callus multiplication on the CM medium, the callus was transferred to the RX medium dedicated to *Fagopyrum* cultures. Once PECCs formed on the callus, they were transferred to the regeneration medium (Supplementary Table S1, sheet 1) and subcultured every two weeks. Shoot-like structures were transferred to a rooting medium without plant growth regulators [64].

According to Grzebelus et al. [18], protoplast viability was estimated just after their isolation as the number of protoplasts with apple-green fluorescence per total number of calculated cells (× 100). Plating efficiency was estimated in 10-day-old protoplast-derived cultures and expressed as the number of cell colonies after cell mitotic divisions per total number of observed undivided cells and cell colonies. Microscopic observations were conducted using an Olympus IX81 inverted fluorescence microscope equipped with a filter set suitable for FDA detection (λ_Ex_ = 475 nm, λ_Em_ = 530 nm). Three independent protoplast isolations were performed, with each treatment represented by three Petri dishes, each of which was considered to be a biological replicate. Observations were made on 100–150 cells per Petri dish. Mean values and standard errors were calculated, and the data were analysed using one-way analysis of variance (ANOVA) with Statistica 13 (TIBCO Software Inc.). Tukey’s *post-hoc* test was applied to identify significant differences between the means.

### Flow cytometry of protoplast-derived plants

Flow cytometry was used to determine the ploidy level of the regenerated *Fagopyrum* plants (Table 1). Fresh and vital leaves from the regenerants and the internal standard *Solanum pseudocapsicum* (2 C DNA = 2,59 pg) [53], were chopped together in a Petri dish in 400 µL of a nuclei extraction buffer (CyStain UV Pr, 05–5002, Sysmex) using a razor blade. The nuclei suspension was filtered through a 30 µm mesh (CellTrics, Sysmex) and stained with DAPI (CyStain UV Precise P, 05–5002, Sysmex) according to the manufacturer’s instructions. The samples were incubated for 5 min in the dark and then analysed using a flow cytometer (CyFlow Space, Sysmex) equipped with 365 nm UV LED as the light source. 4000 nuclei were analysed for each sample.

**Table 1:** Regeneration ability of protoplast-derived callus of *F. tataricum* on culture media supplemented with phenolic inhibitors (AOP, AOPP, OBHA, AIP) and adsorbent (PVP) and ploidy level of regenerated plants

Medium variant	Concentration	Plating efficiency (%) (Mean ± SE)	Regeneration ability	Ploidy level^*^
Control		33.8 ± 14.6^abc^	+	diploid
AOA	1 mM	–	–	–
	3 mM	–	–	–
	5 mM	–	–	–
	10 mM	–	–	–
AOPP	0.2 mM	25.2 ± 8.6^a^	+	tetraploid (25%) diploid (75%)
OBHA	10 µM	44.0 ± 6.8^c^	+	tetraploid (50%) diploid (50%)
	50 µM	27.9 ± 7.8^ab^	–	–
AIP	10 µM	40.5 ± 3.5^bc^	+	diploid
	100 µM	30.0 ± 1.5^ab^	–	–
PVP	1%	33.3 ± 2.2^abc^	+	diploid
	3%	–	–	–

^abc^ Means followed by the same letters were not significantly different at *p* ≤ 0.05

– not able to regenerate, no obtained plants

^* ^Based on relative DNA content from flow cytometry

### Effect of PAL inhibitors and phenolics adsorbents on callus regeneration

A MC obtained from the immature zygotic embryo of *F. tataricum* was used to evaluate the impact of AIP and PVP additives. For plant regeneration, PECCs from the surface of *F. tataricum* callus were transferred onto the regeneration medium (Supplementary Table S1, sheet 1) supplemented with PVP (1 or 3%) or AIP (10 or 100 μM), while medium without any additive was used as control. The PECCs were incubated for seven days in a light chamber at 28 ± 2 °C, 16/8 h (light/dark) photoperiod, and 55 μmol m^−2^ s^−1^ light intensity. Due to the small size of an individual PECC, the mass was determined by grouping ten PECCs, and then the average mass was calculated. On day zero and after seven days on the regeneration medium, the callus derived from the PECCs was individually weighed. The statistical differences were analysed using Statistica ver. 14.0.0.15, using one-way Analysis of Variance (ANOVA) and a *p*-value of < 0.05. Seven-day-old callus of *F. tataricum* cultured on regeneration medium supplemented with PVP (1 or 3%) or AIP (10 or 100 μM) were used for total phenolics analyses, metabolomics, and gene expression analysis.

The calli were kept on the regeneration medium for three weeks, and then the regenerants were transferred onto the rooting medium (Supplementary Table S1, sheet 1), maintaining the same concentration of additives.

### Total phenolics and total flavonoids analyses

Total phenolic and total flavonoid contents in the 7-day-old callus of *F. tataricum* were determined spectrophotometrically using the Folin–Ciocâlteu and Aluminium Chloride colourimetric methods respectively [10, 50]. Around 100 mg of tissue was homogenised in a mortar with liquid nitrogen, resuspended in 2 ml cold 95% MeOH, and incubated for 24 h in the dark at room temperature (RT). The samples were centrifuged at 13,000 g for 5 min at RT, twice. For total phenolics analysis, 100 μl of the supernatant was dissolved in 200 μl of Folin–Ciocâlteu reagent and 800 μl of 700 mM of Na_2_CO_3_. This mixture was incubated for 2 h at RT, centrifuged at 13,000 g for 5 min, and the absorbance was measured at 765 nm in an Eppendorf BioSpectrometer®. Gallic acid was used as the standard, and the results were expressed as millimolar of gallic acid (GA). For total flavonoids analysis, 600 μl of the supernatant was dissolved in 600 μl of AlCl_3_ and incubated for 1 h at RT. The absorbance was measured at 420 nm. Quercetin was used as the standard and the results were expressed as millimolar of quercetin (Q). The data presented are an average of five measurements per treatment.

### Metabolomics

Around 100 mg of 7-day-old callus cultured on different variants of regeneration medium were frozen, and metabolites from grounded material were extracted using a single-phase extraction solution (chloroform/methanol/water, 1:3:1, *v*/*v*/*v*). Frozen samples were homogenised and mixed with 1 mL of the extraction solution for 30 min at 4 °C. Further, the samples were centrifuged for 1 min at 4 °C and 200 µL of sample were lyophilised and resuspended in 50% (*v/v*) methanol for further analysis. Metabolite fingerprinting was performed by FIE-HRMS using a Q Exactive Plus Hybrid Quadrupole Orbitrap Mass Analyser with an Acella UHPLC system (Thermo Fisher Scientific). The *m*/*z* (mass-ion) values were generated in both positive and negative ionisation modes, as described by Baptista et al. 2018. The obtained data are provided in Supplementary Table S1 (sheets 2 and 3). Multivariate analysis was performed using MetaboAnalyst 6.0 (https://www.metaboanalyst.ca/MetaboAnalyst/home.xhtml). Data were log_10_-transformed, and Pareto scaled to the total ion count. The significance of the cross-validated *p*-values, based on the one-way analysis of variance (ANOVA), was set to *p* < 0.05. The multiple comparison and post hoc analysis used Fisher’s LSD. Identification was based on the MS peaks to pathway algorithm (tolerance = 5 ppm, reference library; *Gossypium hirsutum*).

### Chromatography

Extracts were prepared by vigorously vortexing ~ 10 mg of freeze-dried ground callus (cultivated for seven days on various regeneration media) in 1 ml of 80% methanol for 30 min at room temperature. The homogenates were then centrifuged (15 000 × *g*, 10 min, 4 °C) to remove callus debris. The resulting supernatants were stored in the dark at –20 °C.

Phenolic compounds (except gallic acid) were identified using high-performance liquid chromatography (HPLC). For that, Shimadzu LC–10 AS chromatograph equipped with a C18 RP chromatographic column (LiChrospher® 100 RP- 18, 250–4, 5 µm, Merck) and SPD- 10 AV UV–Vis detector was used. The signal detection was set at the wavelengths of 265 and 325 nm. Chromatographic separation was carried out at 30 ± 1 °C using the following solvents: (A) water with acetic acid (0.1%), (B) methanol with acetic acid (0.1%) and applying the gradient: 90% A, 10% B for 20 min; 75% A, 25% B for 30 min; 65% A, 35% B for 40 min; 55% A, 54% B for 50 min; 50% A, 50% B for 60 min; 30% A, 70% B for 62 min; 100% B to 80 min; 80% A, 10% B up to 85 min. The flow rate was 1 mL/min. The analyses were performed in a minimum of four replicates for each callus and analytical standard sample.

A Shimadzu LC–20 AD chromatograph (HPLC) equipped with a C18 RP chromatographic column (Purospher® STAR, 5 µm, Merck) and the Shimadzu SPDM–20 A–DAD photodiode–array detector was used to identify gallic acid. The signal detection was set at the wavelengths of 265 and 325 nm. Chromatographic separation of calli extracts and gallic acid were performed at 33 °C using a mixture of solvents: (A) 10% methanol in water with acetic acid (0.1%), (B) 50% methanol in water with acetic acid (0.1%), and (C) 100% methanol with acetic acid (0.1%). The solvent gradient conditions were as follows: 100% A for 20 min, then increasing the concentration of solvent B to 100% at 55 min of separation, and solvent C concentration to 100% at 75 min of separation (kept until 81 min). The final step was increasing the concentration of solvent A to 100% at 90 min of separation. The analyses were performed in a minimum of three replicates for each callus and the standard sample.

The identification of phenolics was based on the retention times of analytical standards of syringic acid (ChromaDex), sinapic acid (Fluka), chlorogenic acid, *trans*-cinnamic acid, caffeic acid, ferulic acid (ICN Biomedicals), gallic acid, vanillic acid, (+)-catechin, (–)-epicatechin, rutin, and quercetin (Sigma – Aldrich). The standards were solved in 80% methanol and kept in the dark at –20 °C. Water and methanol were of HPLC gradient grade purity (Sigma–Aldrich, Chromasolv for HPLC) and acetic acid were of analytical grade purity.

### Reverse Transcription-Quantitative Polymerase Chain Reaction (RT-qPCR)

The RNA was isolated from 7-day-old callus cultured on different variants of regeneration medium. The callus from media without any additives was used as a control. The RT-qPCR was performed according to the protocol described in Sala-Cholewa et al. [47]. Briefly, the RNA was isolated using FastPure Plant Total TNA Isolation Kit—Polysaccharides and polyphenolics-rich (Vazyme Biotech) and Maxima H Minus First Strand cDNA Synthesis Kit (Thermo Fisher Scientific) and oligo-dT primers were utilised to generate cDNA after removal of DNA from RNA samples. The reactions were performed using a LightCycler® 480 SYBR Green I Master (Roche). The control genes, SAND and ACTIN were used as a reference for the calculation of relative expression level using 2^–∆∆CT^, where ∆∆C_T_ represents ∆C_T_^reference condition^ − ∆C_T_^compared condition^. The two-way ANOVA (*p* < 0.05) followed by Tukey’s honestly-significant-difference test (Tukey HSD-test) (*p* < 0.05) was used to identify any significant differences between the experimental combinations. For the primers designee *F. tataricum* “Pinku1” (GWHBJBL00000000) references genome was used (Chinese National Genomics Data Center database, https://bigd.big.ac.cn/) [19].

## Results

### Protoplast-derived cultures

Protoplasts isolated from two lines of *F. tataricum* showed high viability, ranging between 70–80%, with no significant differences in plating efficiency between the two lines. Plating efficiency across most medium variants ranged from 25–44% (Table 1), except for those supplemented with AOA and 3% of PVP, where no cell division or protoplast culture development was observed. The protoplast medium supplemented with 0.2 mM AOPP displayed a lower (25.2%) plating efficiency than the control (33.8%), while the medium containing 10 µM OBHA resulted in the highest number of cell colonies (44%), although the observed differences were not statically significant.

Upon callus multiplication, PECCs formed in protoplast culture medium variants supplemented with 0.2 mM AOPP (Fig. 1C), 10 µM OBHA (Fig. 1D), 10 µM AIP (Fig. 1F), and 1% PVP (Fig. 1H). However, tissue derived from variants like 50 µM OBHA (Fig. 1E) and 100 µM AIP (Fig. 1G) formed callus-like tissue lacking PECCs. The addition of AOA and 3% PVP resulted in inhibition of protoplast-derived cell division and further development. After ten days on the regeneration medium, somatic embryos (Fig. 1I) were observed. Within two weeks, multiple shoots emerged (Fig. 1J) and after two months, both structures successfully developed into fully matured plants (Fig. 1K). Flow cytometry was used to investigate the effect of phenolic inhibitors on the ploidy of regenerated plants. Obtained data showed a mix of ploidy levels in regenerated plants depending on the treatment. Based on relative DNA content, diploid and tetraploid plants were identified. In the 0.2 mM AOPP variant, 25% of the regenerated plants were tetraploid, while 75% were diploid (Table 1), and for 10 µM OBHA, 50% of the plants were tetraploids (Table 1, Supplementary Figure S3). Media with 10 µM AIP and 1% PVP produced PECCs that regenerated exclusively diploid plants (Table 1).

### Macroscopic observations of morphogenesis

**Fig. 2:**
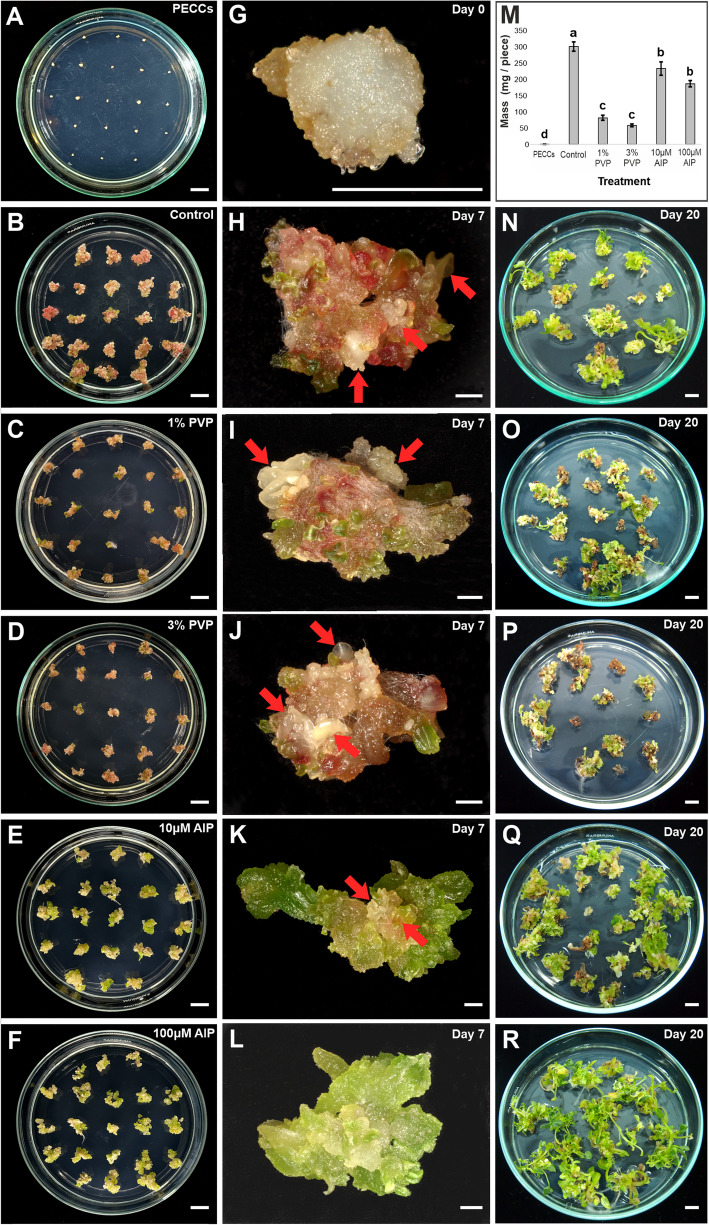
Macroscopic observations of *F. tataricum* PECCs and morphogenesis. **A**–**F** General view of the Petri dishes, **G** closer view of PECCs on day zero and **H**–**L** derived calli after seven days of culture on regeneration medium **B**, **H** without supplements (Control) and **C**, **I** supplemented with 1% PVP, **D**, **J** 3% PVP, **E**, **K** 10 μM AIP and **F**, **L** 100 μM AIP. **M** PECCs mass on day zero and callus mass after seven days on the different variants of regeneration medium. **N**–**R** Shoots/shoot-like structures after 20 days on rooting medium **N** without supplements (control) and supplemented with **O** 1% PVP, **P** 3% PVP, **Q** 10 μM AIP and **R** 100 μM AIP. Red arrows indicate somatic embryos. Scale bar: 1 cm (**A**–**F**; **N**—**R**), 1 mm (**G**–**L**). Abbreviations: AIP – 2-aminoindan- 2-phosphonic acid; PECCs – pro-embryogenic cell complexes; PVP – polyvinyl pyrrolidone

After seven days on regeneration media supplemented with phenolic inhibitors/adsorbents, significant differences were observed in the appearance and mass of callus derived from PECCs (Fig. 2A, G, M). The control callus, grown on medium without additives, was the largest and heaviest, demonstrating the development of somatic embryos (Fig. 2B, H, M; somatic embryos marked by red arrows—H). However, it also displayed extensive browning and dark red discolouration. In contrast, callus supplemented with PVP showed some brownish or dark red spots and developed abundant somatic embryos, but its size and mass varied significantly compared to the control (Fig. 2C, D, J, I, M). AIP-treated callus appeared greener and larger than the PVP-treated one, although its mass was lower than the control (Fig. 2E, F, K, L, M; somatic embryos marked by red arrows—K). Notably, the callus treated with 10 µM AIP had small brownish areas (Fig. 2E, K), while that treated with 100 µM AIP was entirely green and free of discolouration (Fig. 2F, L). After 20 days in the rooting medium, the difference in colouration and shoot and root development remained evident in favour of the treatment with 100 μM of AIP (Fig. 2N-R). In contrast, both treatments with PVP showed poor shoot growth and caused the death of some calli (Fig. 2O, P). In all cases, regeneration primarily occurred via somatic embryogenesis (Fig. 2H–K; somatic embryos marked by red arrows). The flow cytometry analysis showed that all plants regenerated on a rooting medium containing either PVP or AIP were diploids (Table 2, Supplementary Figure S4).

**Table 2:** The ploidy levels of *F. tataricum* regenerants derived from PECCs, which originated from MC cultivated on regeneration media with or without AIP and PVP

Medium variant	Concentration	Ploidy level^a^
Control		diploid
AIP	10 µM	diploid
	100 µM	diploid
PVP	1%	diploid
	3%	diploid

^a^Based on relative DNA content from flow cytometry

### Total phenolics and total flavonoids analyses

**Fig. 3:**
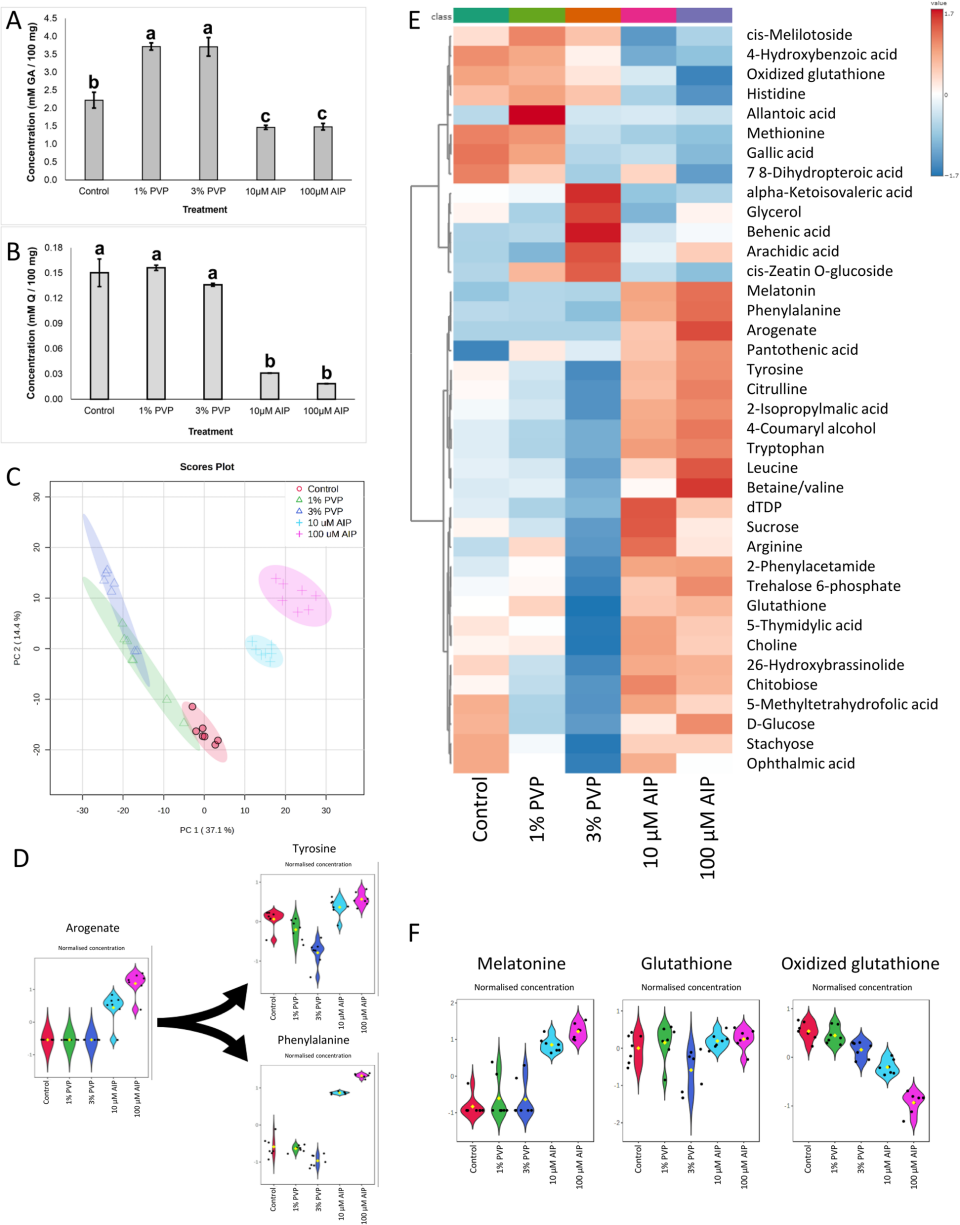
Changes in the metabolomics in the callus of *F. tataricum* after seven days on regeneration medium supplemented with PVP (1 or 3%) or AIP (10 or 100 μM). **A** Total phenolics concentration expressed in millimolar of gallic acid per 100 mg of tissue (mM GA/100 mg of callus), **B** total flavonoids concentration expressed in millimolar of quercetin per 100 mg of tissue (mM Q/100 mg of callus); **C** Principal component analysis scores plot of metabolome distribution of control, PVP and AIP-treated callus; **D** The violin plots of normalised concentrations of arogenate, phenylalanine, and tyrosine; **E** The heatmap of major differentially accumulated metabolites; **F** The violin plots of normalised concentrations of melatonin, glutathione, and oxidized glutathione. Abbreviations: AIP – 2-aminoindan- 2-phosphonic acid; GA – gallic acid; PVP – polyvinyl pyrrolidone, Q – quercetin

Total phenolic and flavonoid concentrations in the calli varied significantly across treatments (Fig. 3A, B). In the case of total phenolics analysis, the control group had 2.2 mM GA per 100 mg of calli. No significant differences were observed between the treatments with the same additive, 1% versus 3% PVP or 10 µM versus 100 µM AIP. However, significant differences were found when comparing different additives. The highest phenolic levels were observed in PVP-treated callus, both showing 3.7 mM GA per 100 mg — a 65% increase over the control. In contrast, AIP-treated calli had 1.5 mM GA, 33% lower than the control (Fig. 3A). The total flavonoid content accounted for 1–7% of the total phenolics in 7-day-old calli. The flavonoids production in the control group represented 6.76% of the total phenolics, while in the treatment with 1% and 3% PVP, and 10 µM and 100 µM AIP, total flavonoids represented 4.2%, 3.67%, 2.13% and 1.25% respectively. No significant differences were observed between the control and both PVP treatments, with flavonoid concentrations ranging from 0.13 to 0.15 mM Q per 100 mg of callus. In contrast, both AIP-treated calli exhibited significantly lower flavonoid concentrations, ranging from 0.018 to 0.031 mM Q per 100 mg of callus, representing an approximately 80% decrease compared to the control (Fig. 3B).

### Metabolomics

Untargeted metabolomics revealed global changes in the callus in response to PVP and AIP treatments. Principal component analysis showed clear separations between the control and treated groups, primarily along PC1, accounting for 37.1% of the variation (Fig. 3C). Differences were also observed between the PVP and AIP treatments. Additionally, PC2, which explained 14.4% of the variance, indicated a dose-dependent effect, with higher concentrations of PVP and AIP leading to greater metabolic variation. However, the differences were more pronounced for AIP than for PVP. The sources of variation between treatments were identified using ANOVA (*p* < 0.05) followed by a Fisher’s LSD post hoc test, to indicate 15,328 significantly different *m/z* (mass-ions). Key statistically significant metabolites linked to the phenylalanine, the substrate for PAL, were selected (Fig. 3D, E, and F). Specifically, melatonin was detected in the AIP-treated callus but was absent in the other callus samples. Glutathione (GSH) levels remained consistent across treatments, with a slight decrease observed only in the 3% PVP treatment. In contrast, oxidized glutathione (GSSG) showed a slight decrease in response to PVP and a more pronounced, dose-dependent decrease with AIP (Fig. 3E, F).

The untargeted metabolomics analysis was complemented by HPLC profiling of polyphenolics, including rutin, catechin, and epicatechin, along with *trans*-cinnamic acid (Supplementary Figure S5, Supplementary Table S1, sheet 4). *Trans*-cinnamic acid levels decreased significantly across all treatments, with the control showing 18.76 µg/g of dry mass compared to just 0.48 µg/g in the 100 µM AIP treatment. The analysis also revealed significant differences in polyphenolic compound concentrations across treatments. The control consistently exhibited the highest levels of most compounds, particularly rutin (4185.98 ± 151.61 µg/g), (–)-epicatechin (5674.62 ± 260.04 µg/g) and (+)-catechin (2311.51 ± 476.45 µg/g). In contrast, treatments with 1% and 3% PVP showed reductions in rutin and chlorogenic acid but increased concentrations of syringic, ferulic, and sinapic acids. Notably, AIP treatments resulted in the lowest concentrations for many compounds, especially at 100 µM, where rutin dropped to 21.97 ± 1 µg/g, (–)-epicatechin to 80.46 ± 1.45 µg/g, and (+)-catechin to 6.66 ± 0.76 µg/g, indicating a strong inhibitory effect on polyphenolic synthesis. Overall, PVP enhanced some acids, while AIP significantly decreased overall polyphenolic levels, with 100 µM having a more significant impact.

### Gene expression

**Fig. 4:**
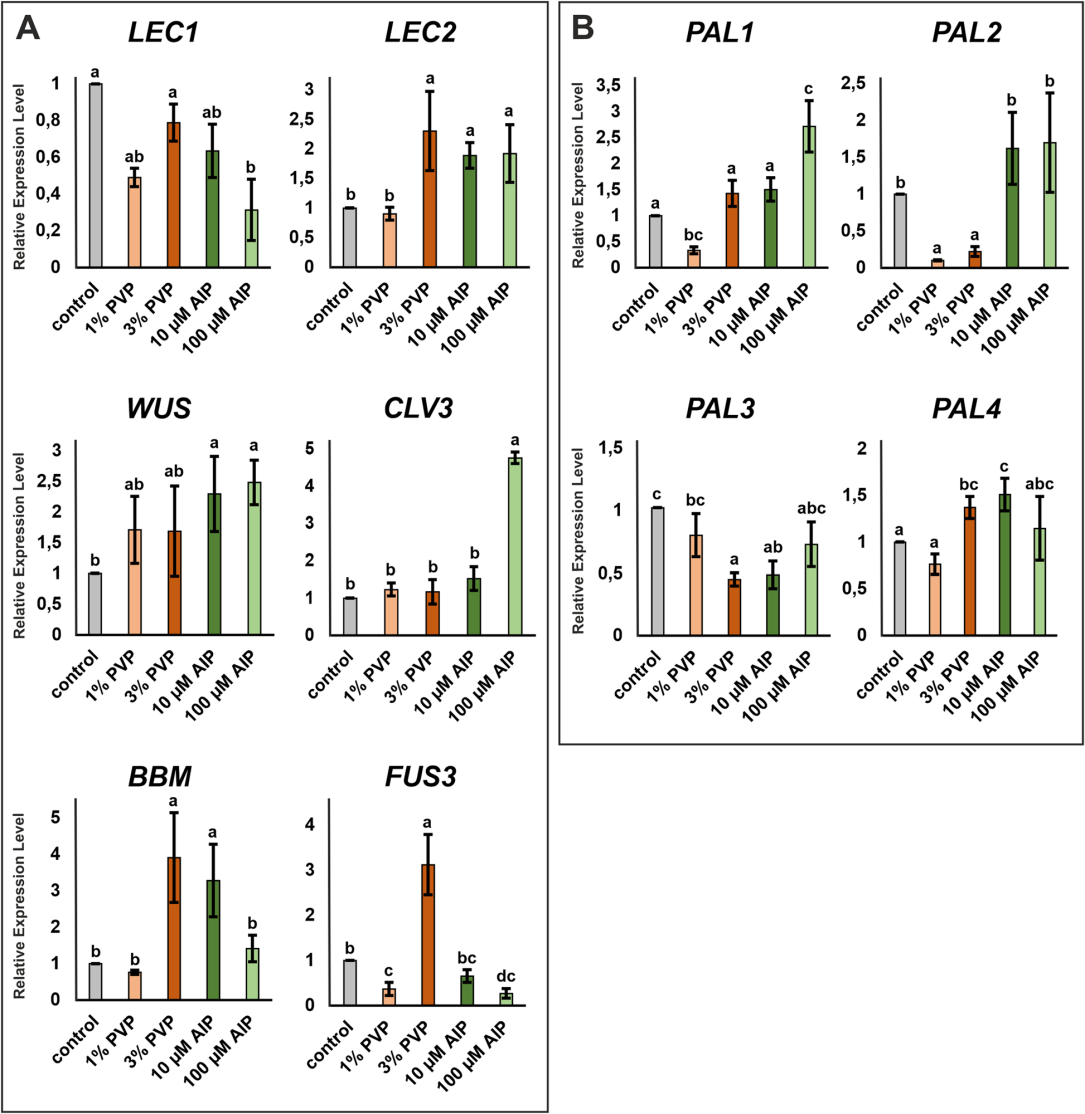
Expression level of **A** transcription factors* (LEC1, LEC2, FUS3, BBM, WUS* and *CLV3)* and **B** phenylalanine ammonia-lyase* (PAL1, PAL2, PAL3* and *PAL4)* genes in 7-day-old callus cultures on regeneration medium supplemented with PVP (1 or 3%) or AIP (10 or 100 μM). The expression level of genes was calibrated to the expression of reference genes, *ACT* (*actin*) and *SAND* (*SAND DNA-binding protein domain*), and to control *F. tataricum* callus. Different letters indicate a significant difference between treatments according to Tukey’s HSD test (*p* < 0.05; *n* = 3; means ± SD are given). Abbreviations: AIP – 2-aminoindane- 2-phosphonic acid; *BBM – BABY BOOM*; *CLV3 – CLAVATA 3*; *FUS3 – FUSCA 3*; *LEC – LEAFY COTYLEDON*, PAL – phenylalanine ammonia-lyase; PVP – polyvinylpyrrolidone; *WUS – WUSCHEL*

Among the key factors involved in somatic embryogenesis, the relevant transcription factor-encoding genes include: *LEAFY COTYLEDON 1* (*LEC1*), *LEAFY COTYLEDON 2* (*LEC2*), BABY BOOM (*BBM*), *WUSCHEL* (*WUS*), *CLAVATA* (*CLV3*), and *FUSCA 3* (*FUS3*) [66]. Given that AIP and PVP influence development and regeneration, we aimed to investigate how these treatments affect the expression of these genes. The analysis of *LEC1*, *LEC2*, *BBM*, *WUS*, *CLV3*, and *FUS3* revealed differential expressions in response to PVP and AIP treatments compared to the control callus (Fig. 4A). We observed that *LEC2* expression increased approximately two-fold in both AIP treatments and 3% PVP compared to the control and 1% PVP, while *LEC1* expression remained relatively stable. The highest *BBM* expression was observed in 3% PVP and 10 μM AIP, with levels 3.9 and 3.3-fold higher than control, respectively. *FUS3* expression was highest in 3% PVP, reaching 3.1-fold higher than control, but was lower in both 1% PVP and 100 μM AIP, with 2.7-fold and 3.7-fold decreases, respectively. *WUS* expression was significantly higher in AIP treatments, approximately 2.4 times that of the control, while PVP treatments showed no statistically significant difference compared to the control. *CLV3* expression remained stable across treatments, except for a peak in response to 100 μM AIP, which was 4.8-fold higher than the control.

Given that AIP is an inhibitor of PAL, and PAL is a key rate-limiting enzyme in the phenylpropanoid pathway, we investigated the changes in PAL expression. Although PVP does not directly influence PAL, we decided to explore its potential effects [67]. Five genes encoding PAL have been identified in the *F. tataricum* genome [19]. The analysis of *PAL1 to PAL4* showed that their expression remained largely constant across treatments, except for *PAL1* and *PAL2*. The expression of *PAL1* was significantly higher in response to 100 μM AIP, being 2.7 times higher than the control, while it was three times lower in 1% PVP. *PAL2* showed a sharp decrease in response to PVP, with expression levels being 9.9 and 4.5 times lower in 1% and 3% PVP, respectively. No expression of *PAL5* was detected in the callus.

## Discussion

### Protoplast-derived cultures and plant regeneration in response to phenolic inhibitors and PVP

The influence of the phenolic inhibitors (AOP, AOPP, OBHA, AIP) and adsorbent (PVP) on protoplast-derived cultures regeneration varied significantly. AIP at 10 µM concentration promoted culture development and enabled diploid plant regeneration, supporting previous findings in *Cannabis sativa* and *Miscanthus* × *giganteus*, where AIP reduced phenolic synthesis and improved tissue quality and regeneration rates [13, 37]. However, regeneration potential was lost at higher AIP concentrations (100 µM), suggesting that excessive phenolic inhibition may negatively affect further cell colony development and regenerating ability. For American elm tissue, a reduction in flavonoid content and tissue browning was observed, along with an improvement in protoplast isolation frequency, faster cell wall reconstruction, and a quicker onset of the first cell division [23]. PVP, at 1%, positively influenced protoplast-derived cultures regeneration, resulting in regenerated diploid plants, while an increase of PVP to 3% resulted in loss of regeneration potential. Previous studies in grapevine protoplast-derived cultures reported that PVP’s adsorptive capacity reduced oxidative stress and supported cell growth [42]. In contrast, the phenolic inhibitor AOA showed no regenerative potential at any tested concentration, likely due to excessive inhibition of phenolic pathways essential for protoplast survival. Interestingly, AOA inhibits PAL activity in hypocotyls of *F. esculentum* and effectively reduces light-induced anthocyanin formation [20]. Additionally, studies in carnation flowers and tomato plants have shown that AOA inhibits ethylene biosynthesis by blocking the formation of 1-aminocyclopropane- 1-carboxylic acid (ACC), a key ethylene precursor, which may further restrict cell division and regeneration ability [7, 25]. AOPP and OBHA exhibited limited regeneration potential, leading to tetraploid plants forming, which may restrict their application depending on specific tissue culture objectives. Previous studies have shown that AOPP and OBHA effectively inhibit PAL, reducing anthocyanins and lignin content in the cell wall. We could speculate that prolonged exposure to AOPP and OBHA, as seen in protoplast-derived cultures regeneration, may induce polyploidisation, a phenomenon not previously studied in fully grown plants. Notably, AOPP was previously tested for control of the browning of lettuce tissue, aiming to enhance its quality and shelf-life [57]. These findings highlight the importance of optimising compound concentrations to balance phenolic reduction with protoplast viability and successful regeneration of genetically stable plants.

### The response of PECCs isolated from Tartary buckwheat morphogenic calli to AIP and PVP

Based on the observed effects of tested inhibitors and adsorbents, we have further investigated the impact of PVP and AIP on plant regeneration from PECCs isolated from Tartary buckwheat morphogenic calli. Both PVP and AIP had distinct effects on *F. tataricum* callus. PVP’s role in improving callus regeneration is particularly notable in its capacity to reduce phenolic compound accumulation, which often inhibits tissue growth and viability. In *F. tataricum*, 3% PVP significantly boosted the expression of embryogenesis genes such as *BBM* and *FUS3*, supporting callus morphogenesis and later stages of embryo maturation as *FUS3* is a key regulator of seed storage proteins and other reserved materials [62]. Notably, no protoplast and cell colony development was observed when the medium was supplemented with 3% PVP, suggesting that protoplasts are particularly sensitive to higher concentrations of this adsorbent. A similar sensitivity was observed in the cultures supplemented with 100 µM of AIP. The differing responses to higher concentrations of PVP and AIP could be attributed to the distinct conditions experienced by PECCs and developing protoplasts. While PECCs are cultivated on the surface of the medium, protoplasts remain submerged in the medium containing PVP and AIP, potentially exposing them to higher localised concentrations of these compounds. Previous studies have shown that in sugarcane meristem cultures, 0.03% of PVP (in combination with 0.002% diethyldithiocarbamic acid) resulted in a remarkable increase in regeneration rates and survival, achieving 100% survival and an average of 3.8 shoots per explant [43]. Similarly, in teak, PVP treatment accelerated callus emergence and improved shoot length, showing its positive influence on regeneration [60]. B5 medium combined with PVP supported better callus formation in chestnut compared to MS medium [2]. Conversely, AIP primarily inhibits PAL, reducing phenolic synthesis rather than absorbing existing phenolic compounds. In our study, AIP-treated *F. tataricum* callus exhibited lower phenolic levels and a greener appearance, particularly at a concentration of 100 µM. Our findings on AIP-treated *F. tataricum* callus support this, as they showed upregulation of *WUS*, a key gene in stem cell maintenance, suggesting its potential to promote early-stage callus regeneration by sustaining meristematic activity [26].

AIP and PVP significantly affected callus mass, with a reduction observed in PVP-treated callus and a smaller decrease in AIP-treated callus. It may be linked to the distinct mechanisms of action of these compounds. Overall, PVP reduced the contribution of flavonoids to total phenolics, while AIP led to an even greater decrease. Specifically, metabolomic analyses revealed a pronounced reduction in polyphenols, especially rutin, in AIP treatments, while PVP treatments led to a moderate, dose-dependent reduction. The observed production of polyphenols in AIP-treated callus may be attributed to the activity of TAL, which enables the biosynthesis of *p*-coumaric acid from tyrosine, a compound that was notably increased in the treated callus. This observation is consistent with previous studies showing that AIP presence in *Miscanthus* × *giganteus* reduced phenolic content but was not abolished completely (from 2242.34 μg/g to 1569.71 μg/g of dry weight). Simultaneously, the presence of AIP increased regeneration rates up to 58.3% in combination with specific concentrations of 2,4-D [13]. Concurrently, PVP-treated callus showed decreases in amino acids such as tryptophan, leucine, tyrosine, betaine/valine, and phenylalanine, as well as in saccharides (mono- and disaccharides). The overall reduction in callus mass and amino acid and sugar levels could be attributed to PVP’s non-selective binding of nutrients, making them less available to the callus. This could pose a challenge in *F. tataricum* tissue cultures, as PECCs rely on compounds released by the ‘soft’ callus [5, 44]. The compound often contains hydroxyl groups and thus may be adsorbed by PVP. The reduced nutrient availability seems to decrease *PAL2* expression without affecting other PAL genes. In contrast, AIP-treated callus exhibited a notable accumulation of phenylalanine, a substrate for PAL, with no corresponding increase/decrease in PAL gene expression. As AIP is a potent competitive PAL inhibitor, the buildup of phenylalanine without increased PAL activity likely explains the observed effects. Simultaneously, we detected arogenate exclusively in AIP-treated callus, with levels increasing in a dose-dependent manner. In plants, phenylalanine is synthesised from prephenate (part of the shikimate-chorismate pathway), which can be converted into phenylalanine via either phenylpyruvate with subsequent transamination or via arogenate through transamination of prephenate followed by dehydration and decarboxylation. Arogenate can also serve as a precursor for tyrosine synthesis [14]. The observed increase in phenylalanine may have led to a downstream build-up of arogenate, potentially redirecting metabolism toward increased tyrosine biosynthesis. Interestingly, in AIP-treated callus, levels of other amino acids like leucine, tryptophan, and betaine/valine were upregulated, while histidine and methionine levels were downregulated. According to HPLC analysis, cinnamic acid, a product of PAL activity, was significantly reduced in both the PVP and AIP treatments. However, AIP proved to be more effective in decreasing cinnamic acid levels.

We also observed changes in the levels of GSH and GSSG in the treated callus, resulting in a higher GSH/GSSG ratio. This suggests reduced oxidative stress within the cells, particularly in the AIP-treated callus, where the demand for glutathione oxidation was lower. A high GSH/GSSG ratio is often associated with rapid cell growth. For example, endogenous GSSG levels decline in maturing somatic embryos of *Eleutherococcus senticosus* [49]. Applying GSH has also been found to promote the proliferation of embryogenic callus cells in Korean pine during somatic embryogenesis [16]. The decrease in GSSG, particularly in AIP-treated callus, may be linked to the presence of melatonin, which was detected exclusively in these samples. Melatonin is a potent, terminal antioxidant that protects cells without depleting cellular GSH levels [30]. Recent research has shown that melatonin dramatically enhances *Agrobacterium*-mediated transformation efficiency in carnation and doubles shoot regeneration rates [1]. Similarly, in rice callus cultures, melatonin supplementation boosted morphogenesis and increased the expression of antioxidant-related genes [55].

## Conclusions

This study demonstrates the varied effects of phenolic inhibitors (AOA, AOPP, OBHA, AIP) and the adsorbent PVP on the regeneration potential of *F. tataricum* protoplast-derived cultures. AIP at 10 µM proved effective in promoting protoplast and cell colony development and diploid plant regeneration, while 100 µM inhibited regeneration. Similarly, 1% PVP also supported diploid plant regeneration, but regeneration potential was lost at 3% of PVP. Notably, AOPP and OBHA resulted in the regeneration of polypoid plants, limiting their potential use in in vitro cultures.

Further investigation into AIP and PVP effects on PECCs isolated from Tartary buckwheat MC regeneration highlighted their distinct mechanisms. PVP’s ability to adsorb phenolics reduced oxidative stress, leading to nutrient depletion, consequently decreasing callus mass and amino acid availability. In contrast, AIP suppressed phenolic synthesis, increasing the presence of amino acids such as leucine, tryptophan, and betaine/valine. AIP- and PVP-treated callus also showed differences in redox balance with AIP presence, leading to increased GSH/GSSG ratio, which suggests lower oxidative stress. The presence of melatonin in AIP-treated callus likely contributed to this effect, as melatonin acts as an antioxidant, enhancing regeneration and transformation efficiency, as seen in other plant systems. These findings emphasise the importance of carefully balancing phenolic inhibition with nutrient availability and oxidative control for effective *F. tataricum* somatic embryogenesis and plant regeneration.

## Supplementary Information

Supplementary Material 1

Supplementary Material 2

## Data Availability

The datasets supporting the conclusions of this article are included within the article and its additional files.

## Abbreviations

ACC: 1-Aminocyclopropane- 1-carboxylic acid
AIP: 2-Aminoindane- 2-phosphonic acid
AOA: ɑ-Aminooxyacetic acid
AOPP: ɑ-Aminooxy-β-phenylpropionic acid
*BBM*: *BABY BOOM*
*CLV3*: *CLAVATA* 3
CM: Callus multiplication medium
FDA: Fluorescein diacetate
*FUS3*: *FUSCA 3*
GA: Gallic acid
GSH: Glutathione
GSSG: Glutathione oxidised
HPLC: High-performance liquid chromatography
*LEC1*: *LEAFY COTYLEDON1*
*LEC2*: *LEAFY COTYLEDON2*
MC: Morphogenic callus
OBHA: O-benzylhydroxylamine hydrochloride
PAL: Phenylalanine ammonia-lyase
PECCs: Proembryogenic cell complexes
POD: Peroxidase
PP0: Polyphenol oxidase
PVP: Polyvinylpyrrolidone
Q: Quercetin
RT: Room temperature
TAL: Tyrosine ammonia-lyase
*WUS*: *WUSCHEL*
