# Correction: Efect of potent inhibitors of phenylalanine ammonia‑lyase and PVP on in vitro morphogenesis of *Fagopyrum tataricum*

**DOI:** 10.1186/s12870-025-06739-9

**Published:** 2025-05-26

**Authors:** Reneé Pérez-Pérez, Artur Pinski, Magdalena Zaranek, Manfred Beckmann, Luis A. J. Mur, Katarzyna Nowak, Magdalena Rojek-Jelonek, Anna Kostecka-Gugała, Przemysław Petryszak, Ewa Grzebelus, Alexander Betekhtin

**Affiliations:** 1 Institute of Biology, Biotechnology and Environmental Protection, Faculty of Natural Sciences, University of Silesia in Katowice, Katowice, Poland; 2 Institute of Biological, Environmental and Rural Sciences, Aberystwyth University, Penglais Campus, Aberystwyth, Wales SY23 2DA UK; 3 Department of Plant Biology and Biotechnology, Faculty of Biotechnology and Horticulture, Univer- Sity of Agriculture in Krakow, Ave. Mickiewicza 21, 31 - 120 Krakow, Poland

**Correction** **:** **BMC Plant Biol 25, 469 (2025)**

**https://doi.org/10.1186/s12870-025-06440-x**

Following publication of the original article [1], the authors found an error in the published version of Figure 3. Unfortunately, the treatments were labelled incorrectly and should be corrected from Control, 1% PVV, 1% PVP, 10 µM AIP, and 10 AIP to **Control, 1% PVP, 3% PVP, 10 µM AIP, and 100 µM AIP**.

Incorrect Fig. 3

**Fig. 3:**
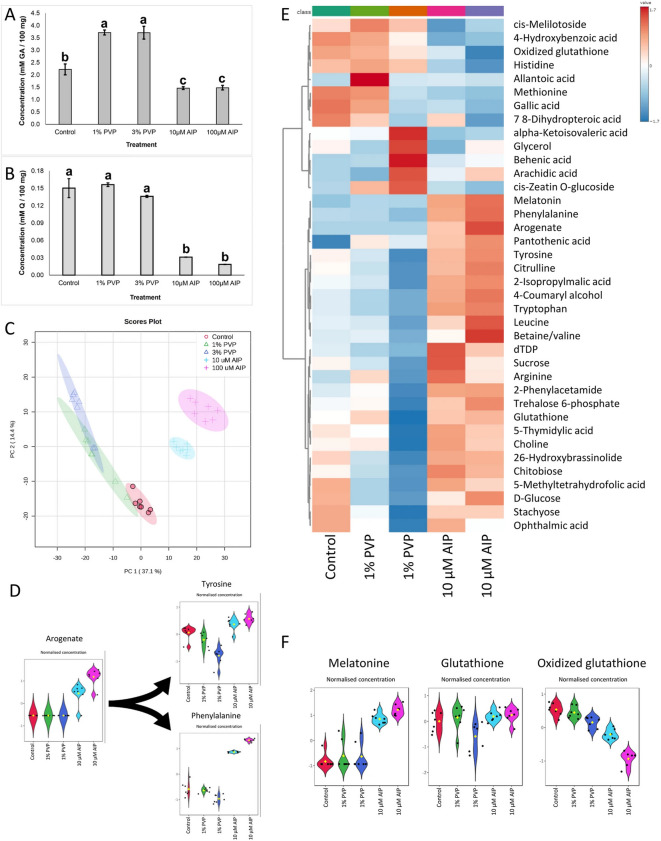
Changes in the metabolomics in the callus of *F. tataricum* after seven days on regeneration medium supplemented with PVP (1 or 3%) or AIP (10 or 100 μM). **A** Total phenolics concentration expressed in millimolar of gallic acid per 100 mg of tissue (mM GA/100 mg of callus), **B** total favonoids concentration expressed in millimolar of quercetin per 100 mg of tissue (mM Q/100 mg of callus); **C** Principal component analysis scores plot of metabolome distribution of control, PVP and AIP-treated callus; **D** The violin plots of normalised concentrations of arogenate, phenylalanine, and tyrosine; **E** The heatmap of major diferentially accumulated metabolites; **F** The violin plots of normalised concentrations of melatonin, glutathione, and oxidized glutathione. Abbreviations: AIP – 2-aminoindan- 2-phosphonic acid; GA – gallic acid; PVP – polyvinyl pyrrolidone, Q – quercetin

**Correct **Fig. 3

**Fig. 3:**
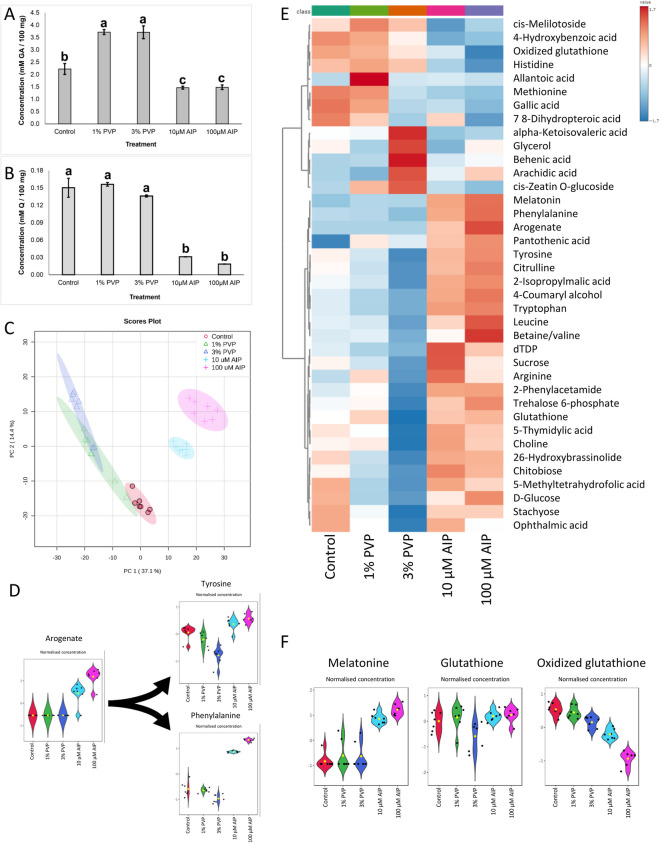
Changes in the metabolomics in the callus of *F. tataricum* after seven days on regeneration medium supplemented with PVP (1 or 3%) or AIP (10 or 100 μM). **A** Total phenolics concentration expressed in millimolar of gallic acid per 100 mg of tissue (mM GA/100 mg of callus), **B** total favonoids concentration expressed in millimolar of quercetin per 100 mg of tissue (mM Q/100 mg of callus); **C** Principal component analysis scores plot of metabolome distribution of control, PVP and AIP-treated callus; **D** The violin plots of normalised concentrations of arogenate, phenylalanine, and tyrosine; **E** The heatmap of major diferentially accumulated metabolites; **F** The violin plots of normalised concentrations of melatonin, glutathione, and oxidized glutathione. Abbreviations: AIP – 2-aminoindan- 2-phosphonic acid; GA – gallic acid; PVP – polyvinyl pyrrolidone, Q – quercetin.

The correction does not compromise the validity of the conclusions and the overall content of the article. The author group has been updated above and the original article [1] has been corrected.

## References

[1] Pérez-Pérez R, Pinski A, Zaranek M, et al. Effect of potent inhibitors of phenylalanine ammonia-lyase and PVP on in vitro morphogenesis of *Fagopyrum tataricum*. BMC Plant Biol. 2025;25:469. 10.1186/s12870-025-06440-x. 40229725 10.1186/s12870-025-06440-x PMC11998252

