# Supplementary material for: Effect of potent inhibitors of phenylalanine ammonia-lyase and PVP on in vitro morphogenesis of Fagopyrum tataricum
Source: BMC Plant Biol. 2025 Apr 15;25:469. doi: 10.1186/s12870-025-06440-x (PMC11998252; doi:10.1186/s12870-025-06440-x)

Supplementary Figure S1. Schematic representation of the Phenylpropanoid Pathway and the inhibition of the Phenylalanine ammonia-lyase (PAL) enzyme by AIP.

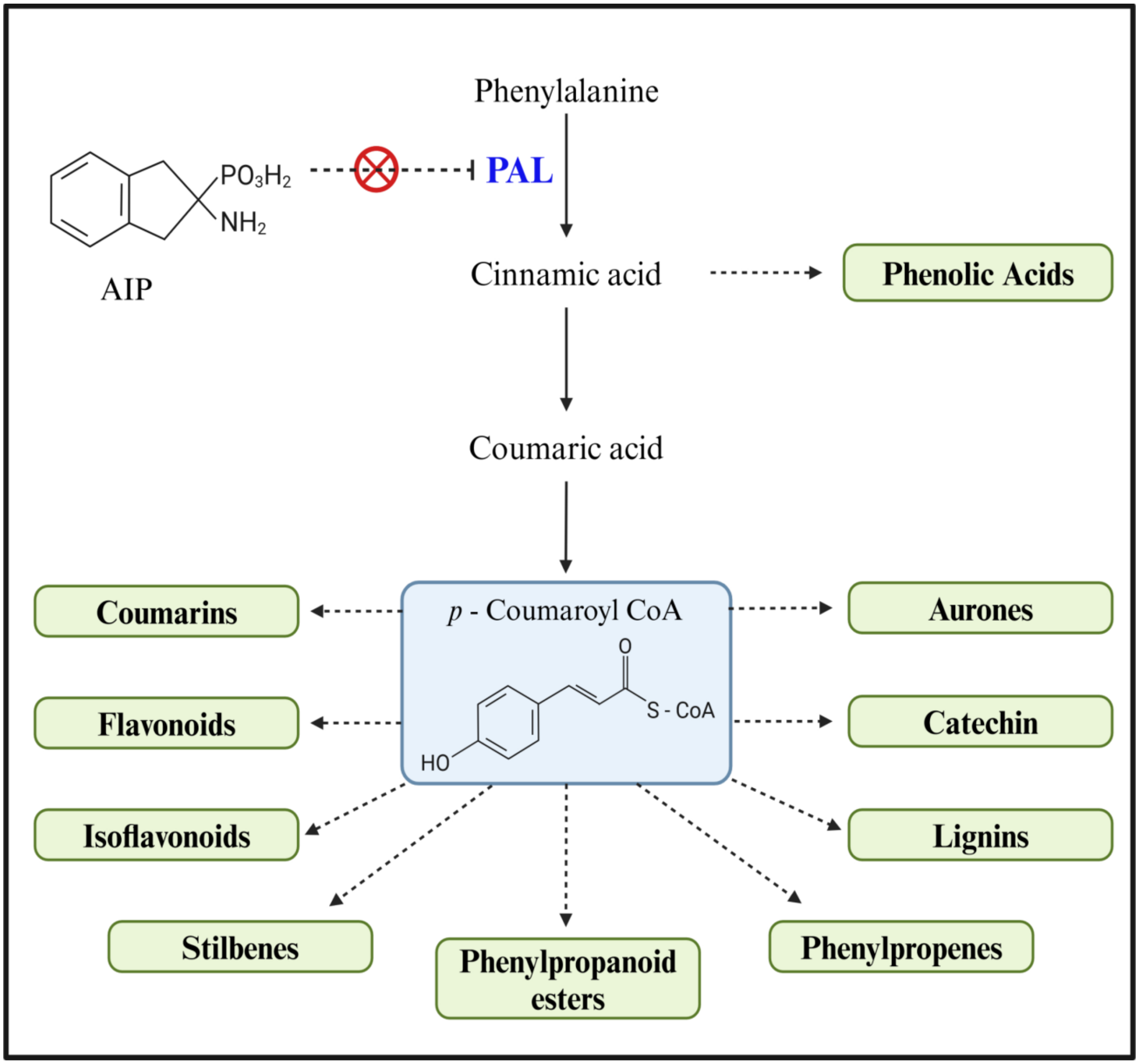

Supplementary Figure S2. Schematic representation of the action mechanism of PVP. (**i**) monomeric unit of PVP, (**ii**) PVP strand and (**iii**) example of adsorption of rutin by PVP strands through hydrogen bonds.

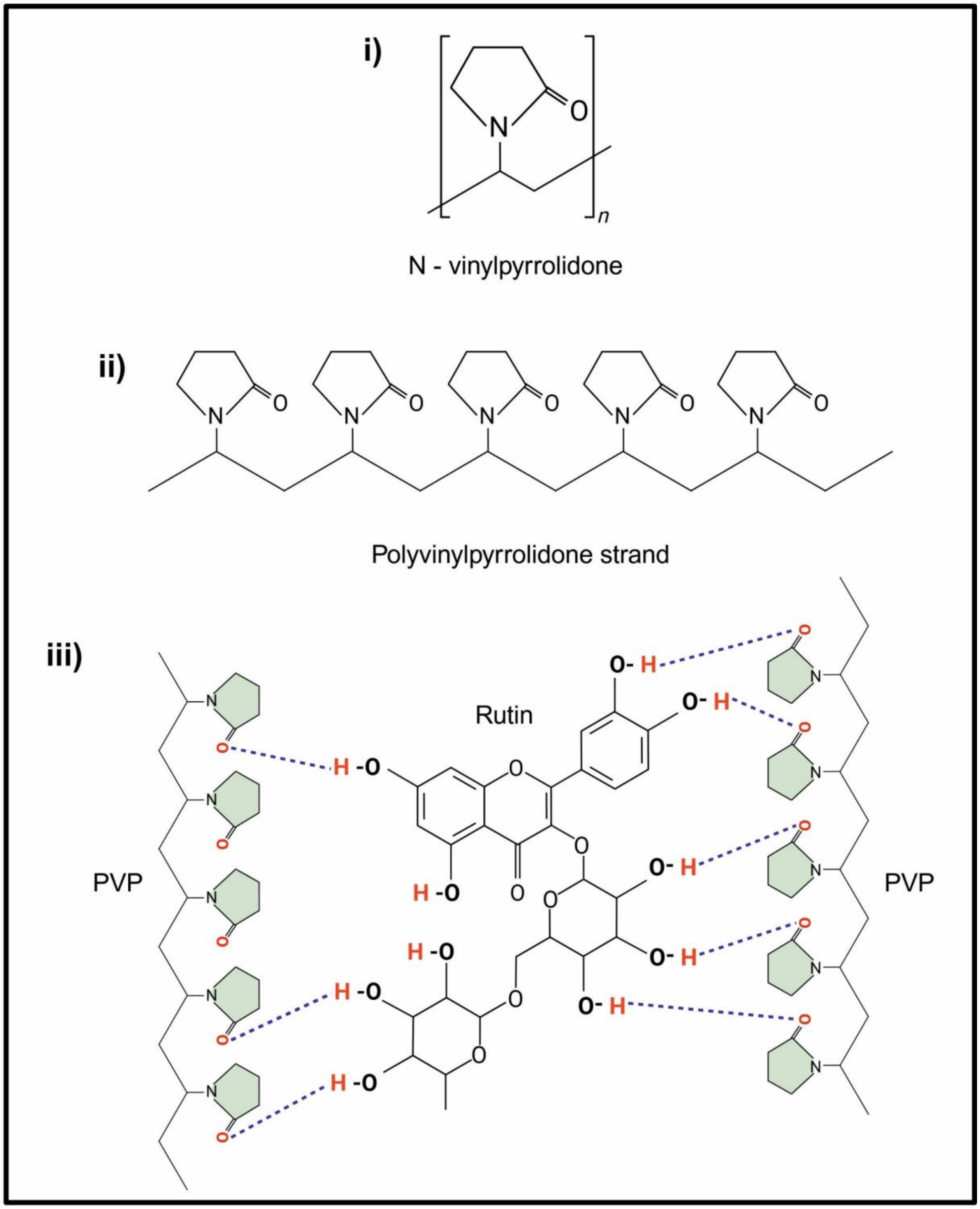

Supplementary Figure S3. Flow cytometer histograms presenting the relative DNA content in the leaves of plant-derived from protoplast cultures of *F. tataricum*. Control plants (**A**), plants derived from protoplast cultivated on culture medium variant with 0.2 mM AOPP (**B**), 10 µM OBHA (**C**), 10 µM AIP (**D**), 1% PVP (**E**).

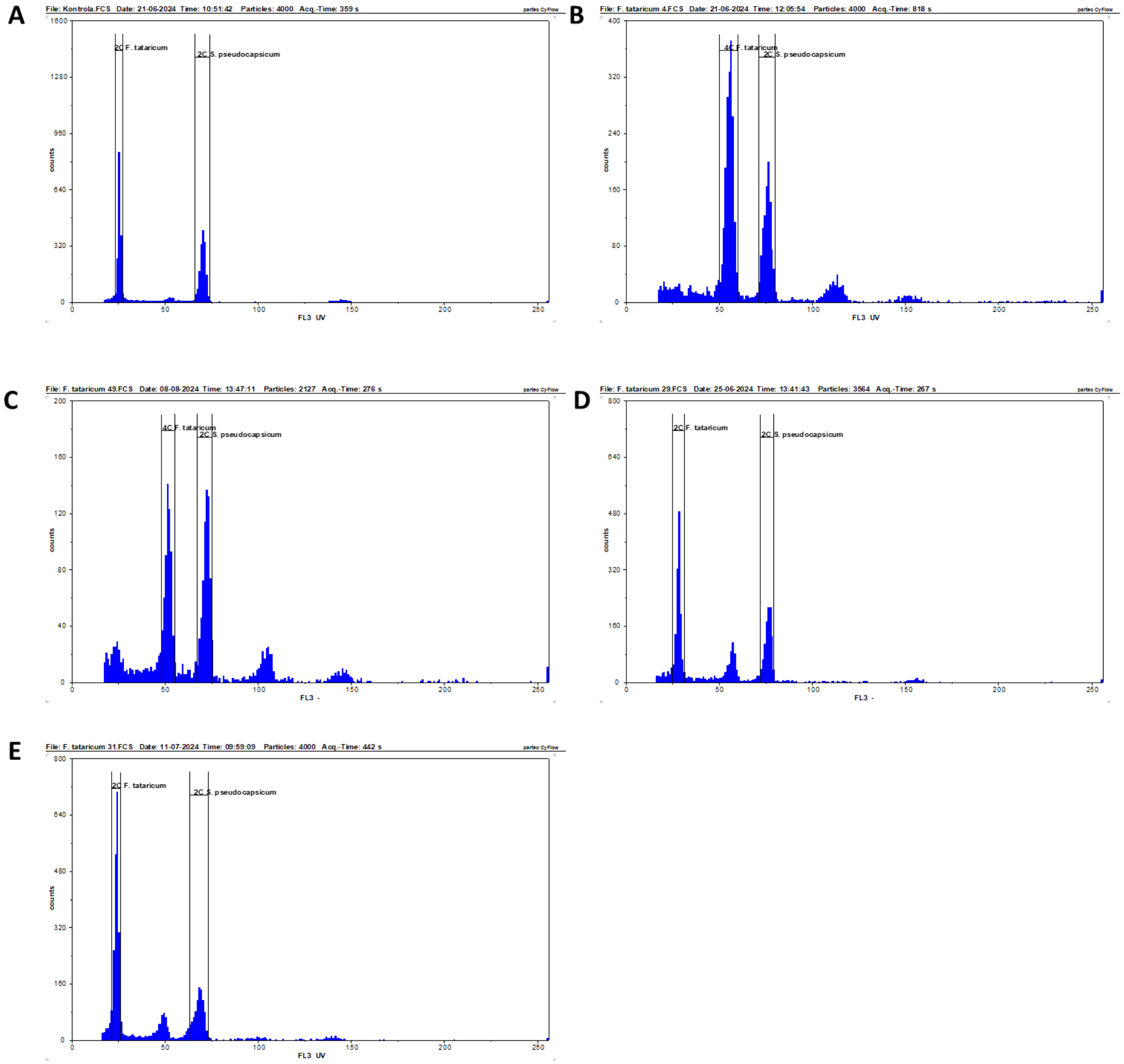

Supplementary Figure S4. Flow cytometer histogram showing relative DNA content in leaves of PECCs-derived plants of *F. tataricum*. The control plants grown from seeds (**A**), PECCs-derived plants cultivated on rooting medium (**B**) and rooting medium supplemented with 1% PVP (**C**), 3% PVP (**D**), 10 μM AIP (**E**), and 100 μM AIP (**F**).

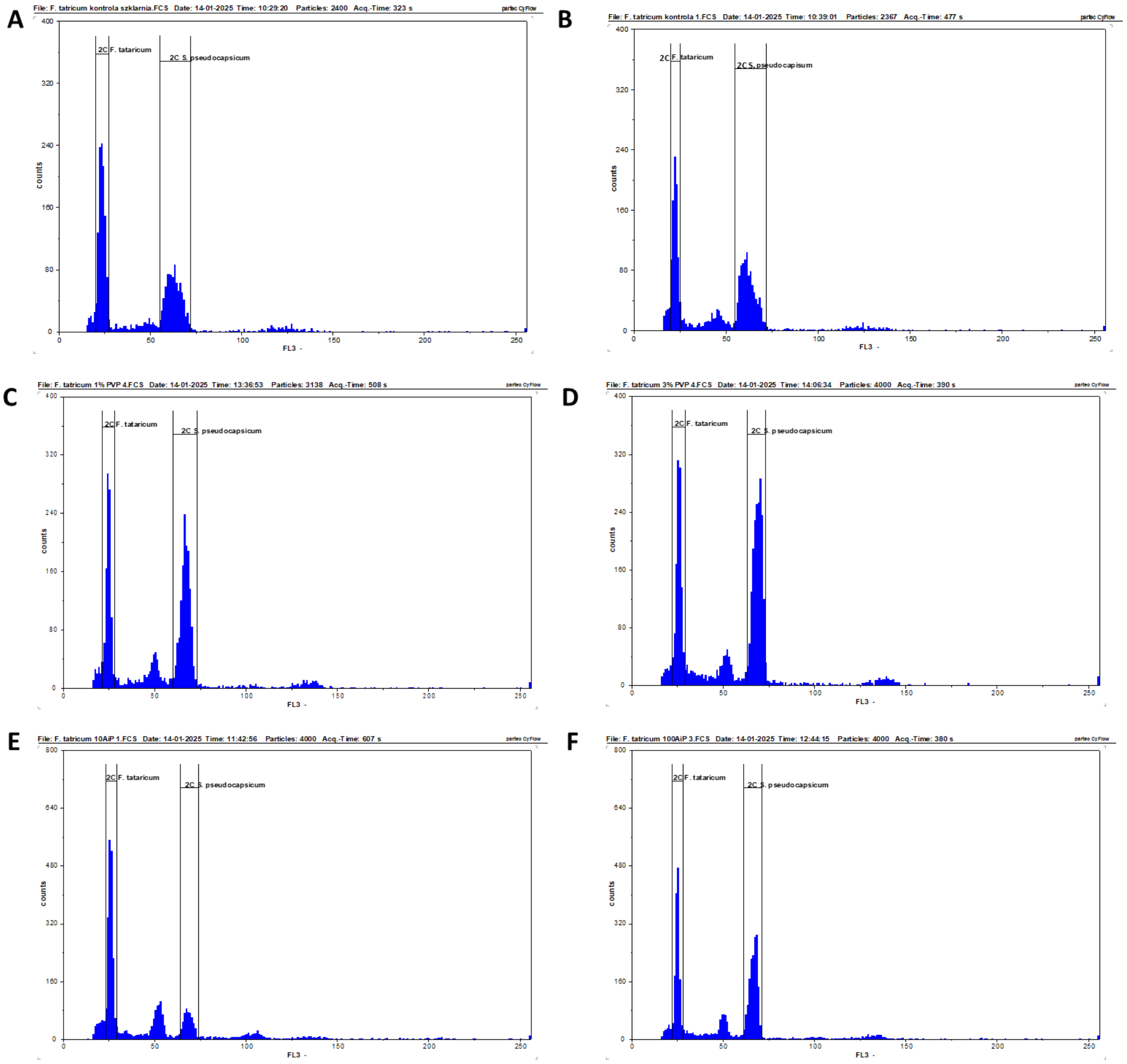

Supplementary Figure S5. Cluster map of HPLC profiling of polyphenolics, including rutin, catechin, and epicatechin, along with trans-cinnamic acid. *AIP – 2-aminoindan-2-phosphonic acid; PVP – polyvinyl pyrrolidone.*

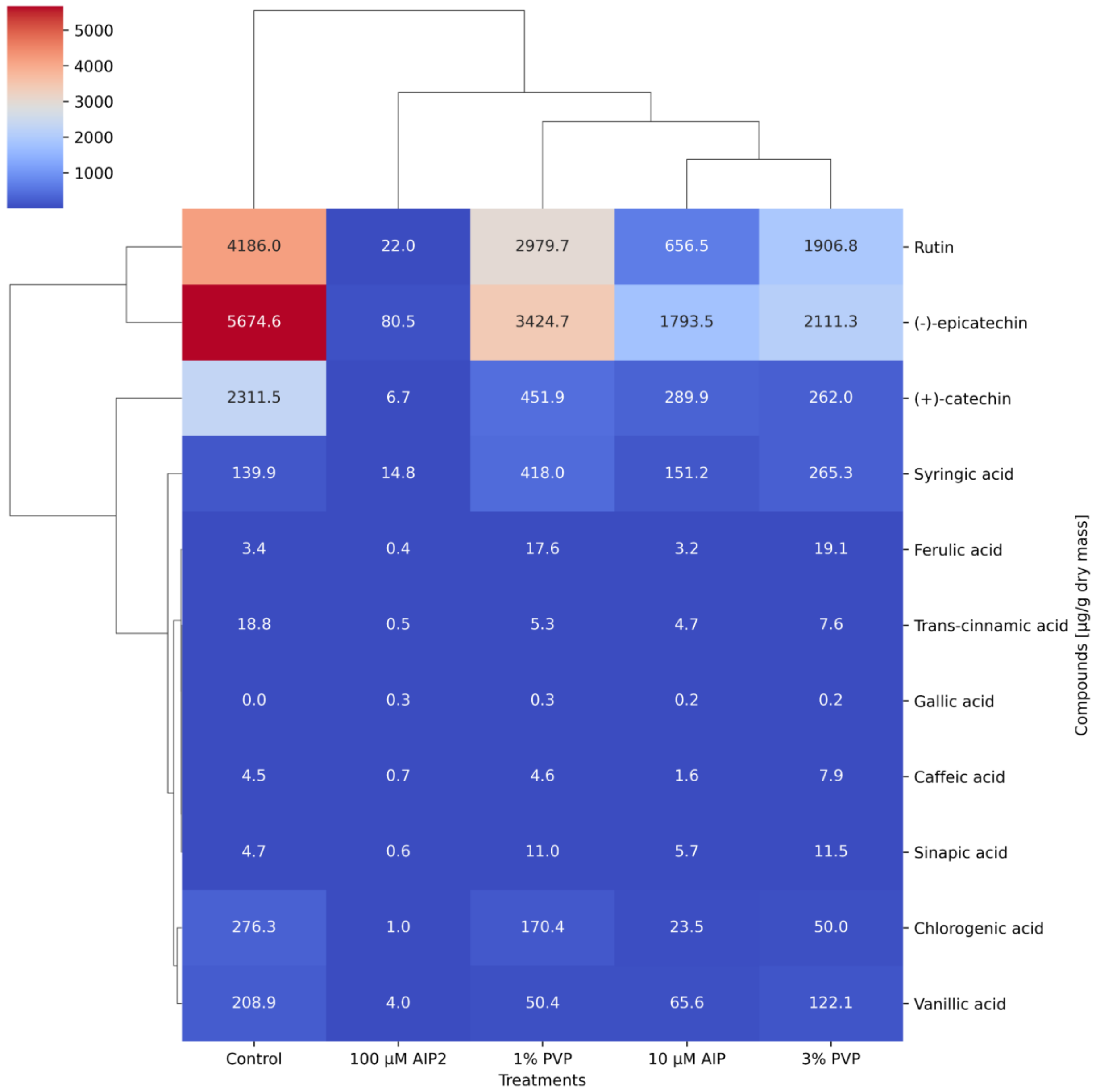

Supplement: Supplementary file 2 — Supplementary Material 2 [file 12870_2025_6440_MOESM2_ESM.pdf]
